# Supplementary figures and images for: Integrated analysis of lncRNA and gene expression in longissimus dorsi muscle at two developmental stages of Hainan black goats
Source: PLoS One. 2022 Oct 31;17(10):e0276004. doi: 10.1371/journal.pone.0276004 (PMC9621442; doi:10.1371/journal.pone.0276004)

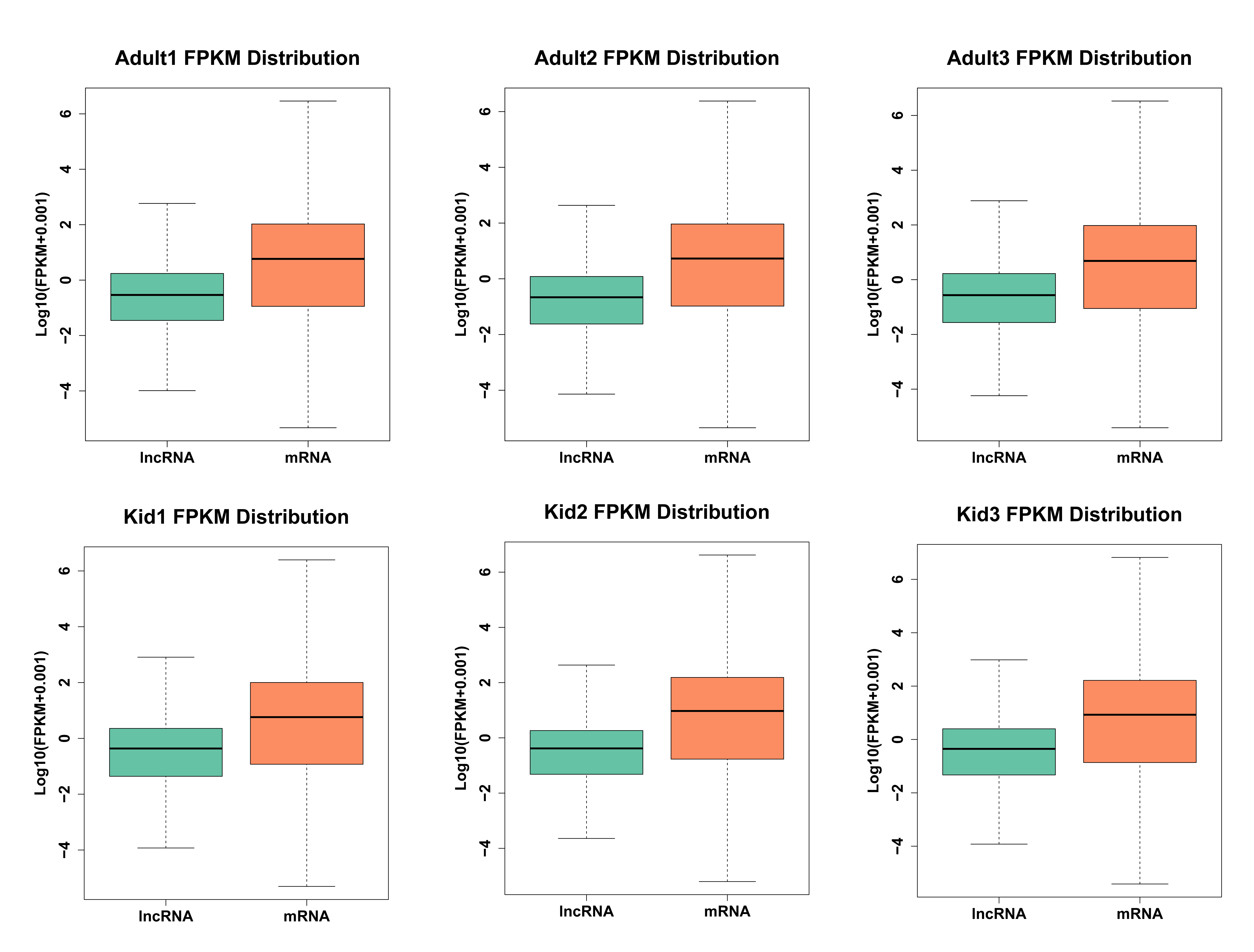

Supplement: S1 Fig — (TIF) [file pone.0276004.s001.tif]

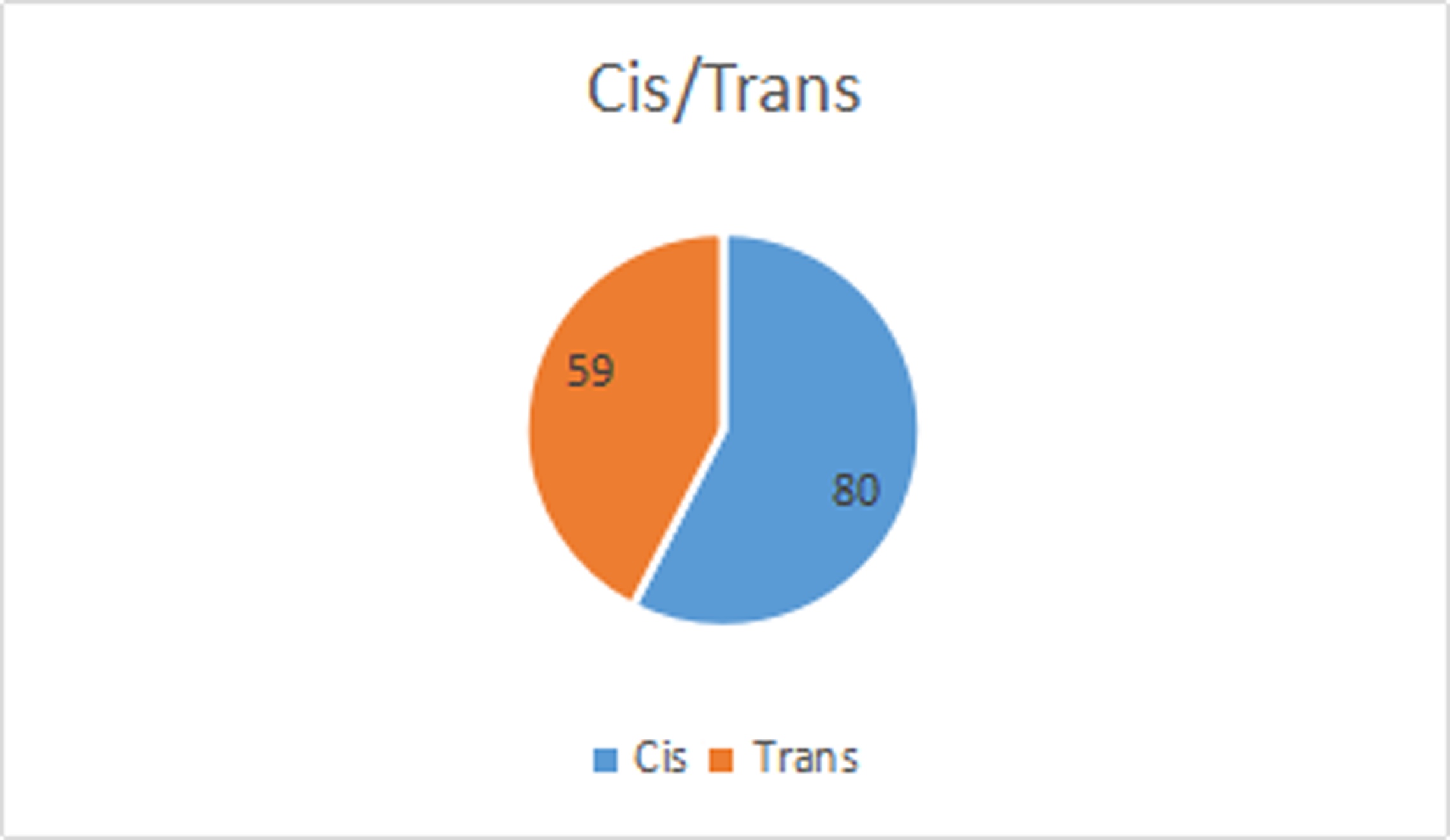

Supplement: S2 Fig — (TIF) [file pone.0276004.s002.tif]
