# Supplementary material for: Integrated analysis of lncRNA and gene expression in longissimus dorsi muscle at two developmental stages of Hainan black goats
Source: PLoS One. 2022 Oct 31;17(10):e0276004. doi: 10.1371/journal.pone.0276004 (PMC9621442; doi:10.1371/journal.pone.0276004)
Supplement: S1 Table — (DOCX) [file pone.0276004.s003.docx]

*Table S1. Premers used in this study*

| LncRNA names | RPKM -Adult | RPKM -Kid | Primer sequences (5' to 3') | Tm (°C ) | Product (bp) |
| --- | --- | --- | --- | --- | --- |
| MSTRG.148339 | 0.45 | 4.49 | F CTCATGTCGTGACCGTCC | 53.8 | 91 |
|  |  |  | R AGTCGCTCAGTCGTGTCC | 52.9 |  |
| MSTRG.160907 | 0.14 | 1.38 | F CCCAGGAGACAAGAAACG | 53.0 | 89 |
|  |  |  | R CTGCTGTAGAAATAGAAGGCC | 54.2 |  |
| MSTRG.72042 | 1.18 | 0.20 | F GTTGCCATTTCCTTCTCC | 52.2 | 80 |
|  |  |  | R TCTTCTCTCTGATGGTCGG | 52.4 |  |
| MSTRG.132175 | 0.40 | 1.08 | F GAAATGACAACCCACTCCAG | 54.8 | 72 |
|  |  |  | R TCAATCACACTCTGCCCTC | 53.1 |  |
| MSTRG.146874 | 0.56 | 0.01 | F GGTTACAGTCCATAGGGTCAC | 53.4 | 92 |
|  |  |  | R AGTTGGGCTCAAGTCTACATC | 5.35 |  |
| β-actin |  |  | F GCTATGTCGCCCTGGATTTC | 55.50 | 98 |
|  |  |  | R CACAGGACTCCATACCCAAGAA | 57.30 |  |
