# Supplementary material for: Integrated analysis of lncRNA and gene expression in longissimus dorsi muscle at two developmental stages of Hainan black goats
Source: PLoS One. 2022 Oct 31;17(10):e0276004. doi: 10.1371/journal.pone.0276004 (PMC9621442; doi:10.1371/journal.pone.0276004)
Supplement: Attachment — Submitted filename: Response to Reviewers.docx [file pone.0276004.s010.docx]

PONE-D-22-12097

Integrated analysis of lncRNAs and genes associated with intramuscular fat in longissimus dorsi muscle at two developmental stages of Hainan black goats

PLOS ONE

**Journal Requirements:**

When submitting your revision, we need you to address these additional requirements.

1. Please ensure that your manuscript meets PLOS ONE's style requirements, including those for file naming. The PLOS ONE style templates can be found athttps://journals.plos.org/plosone/s/file?id=wjVg/PLOSOne_formatting_sample_main_body.pdf andhttps://journals.plos.org/plosone/s/file?id=ba62/PLOSOne_formatting_sample_title_authors_affiliations.pdf

Response: We have changed the style of our manuscript to meet PLOS ONE's style requirements according to http://www.journals.plos.org/plosone/s/file?id=wjVg/PLOSOne_formatting_sample_main_body.pdf and <http://www.journals.plos.org/plosone/s/file?id=ba62/PLOSOne_formatting_sample_title_authors_affiliations.pdf> and marked the modifications in red in the revised manuscript.

2. Please remove the text: "Venous blood was kindly collected to ameliorate the suffering of ducks" from your Ethics Statement.

Response: We make a mistake in our initial manuscript. Therefore, we removed the "Venous blood was kindly collected to ameliorate the suffering of ducks" and changed it to “Goats were slaughtered using the electric shock method followed by jugular vein bloodletting method within 30 seconds to ameliorate their suffering” Line 1767-179 in revised version.

3. Thank you for stating the following financial disclosure:

“This work was financially supported by:

1. Key Research and Development Programs of Hainan Province (Grant no. GHYF2022004), Hanlin Zhou received the award, he designed the study.

2. Central Public-interest Scientific Institution Basal Research Fund for Chinese Academy of Tropical Agricultural Sciences (Grant no. 1630032017034), Tieshan Xu received the award, he analysed the data.”

Please state what role the funders took in the study. If the funders had no role, please state: "The funders had no role in study design, data collection and analysis, decision to publish, or preparation of the manuscript."

If this statement is not correct you must amend it as needed.

Please include this amended Role of Funder statement in your cover letter; we will change the online submission form on your behalf.

Response: The founder, Hanlin Zhou, designed the study and the other founder, TieshanXu, analysed the data, which we added in our cover letter.

4. In your Data Availability statement, you have not specified where the minimal data set underlying the results described in your manuscript can be found. PLOS defines a study's minimal data set as the underlying data used to reach the conclusions drawn in the manuscript and any additional data required to replicate the reported study findings in their entirety. All PLOS journals require that the minimal data set be made fully available. For more information about our data policy, please see http://journals.plos.org/plosone/s/data-availability.

"Upon re-submitting your revised manuscript, please upload your study’s minimal underlying data set as either Supporting Information files or to a stable, public repository and include the relevant URLs, DOIs, or accession numbers within your revised cover letter. For a list of acceptable repositories, please see http://journals.plos.org/plosone/s/data-availability#loc-recommended-repositories. Any potentially identifying patient information must be fully anonymized.

Important: If there are ethical or legal restrictions to sharing your data publicly, please explain these restrictions in detail. Please see our guidelines for more information on what we consider unacceptable restrictions to publicly sharing data: http://journals.plos.org/plosone/s/data-availability#loc-unacceptable-data-access-restrictions. Note that it is not acceptable for the authors to be the sole named individuals responsible for ensuring data access.

We will update your Data Availability statement to reflect the information you provide in your cover letter.

Response: We put the “Data Availability Statement” at the end of the paper, behind of “Acknowledgements” in initial version, where it can’t be found easily. Thus, we moved it to the last paragraph of “Methods and Materials” in revised version (Line 179-182).

**Responses to Reviewers**

**Reviewer #1:**

Short title: LncRNAs and genes related to intramuscular fat of goats, change to: Differential LncRNA and gene expression between young and adult goats

Full title: Change to:Integrated analysis of lncRNA and gene expression in longissimus dorsi muscle of young and adult Hainan black goats

Response: We thanked the reviewer for his/her kind suggestion. Therefore, we changed the short title to “Differential LncRNA and gene expression between young and adult goats” and changed the full title to “Integrated analysis of lncRNA and gene expression in *longissimus dorsi* muscle of young and adult Hainan black goats”.

In this study, lncRNA and gene expression levels in muscle of Hainan black goats at different ages were investigated. Although the intramuscular fat (IMF) content of goats at different ages was different, there were other differences in skeletal muscle of goats at different ages, such as tenderness, water content, protein content, amino acids and fatty acids, meat color, pH, etc. It is not true that goat muscle differs only in intramuscular fat during these two developmental stages. Therefore, the authors need to revise the sentences related to IMF in the full text.

Response:**（1）It is true that there are many differences in skeletal muscle of goats at different ages.** We thank you for your kind reminding. Just as you mentioned, the differences in skeletal muscle of goats at different ages not only include IMF, but also tenderness, water content, protein content, amino acids and fatty acids, meat color, pH, etc. In fact, we had measured some indexes that indicated the differences in skeletal muscle of goats at different ages. We did find IMF and muscle fiber diameter in adult LD muscle were extremely significant higher than that in kid LD muscle (P＜0.01), while shear force in adult LD muscle was significant higher than that in kid LD muscle (P＜0.05).

**(2) The reason why we focus on** **the differences of IMF in skeletal muscle of goats at different ages**. To investigate whether lncRNAs and genes had some effects on the differences in skeletal muscle of goats at different ages, we performed lncRNA-seq and RNA-seq. We found many DEGs and DELs, which suggests lncRNAs and genes might play some roles for the differences in skeletal muscle of goats at different ages. Subsequently, we searched the lncRNAs and genes that affect IMF, muscle fiber diameter and shear force. As a result, we found a number of DELs and DEGs were involved in fat deposition (IMF) which was described detailed in our manuscript. In addition, there were also some significantly enriched pathways that affect muscle fiber diameter and shear force, such as PI3K-Akt signaling pathway, mTOR signaling pathway, MAPK signaling pathway (Fig 3D). However, the number of DELs and DEGs was much less in affecting index than that in affecting IMF. **Thus, we based on the follow reasons to focus on the differences of IMF in skeletal muscle of goats at different ages**. **1) There were a number of the IMF-related genes and pathways. 2) The significant difference of IMF content existed in the LD muscle of goats at different ages. 3) IMF content was significantly correlated with the meat quality of goats. 4) It is difficult that investigate all the differences in skeletal muscle of goats at different ages in one study.** **Based on the considerations above, we only focused on the IMF difference in skeletal muscle of goats at different ages in this paper.**

**(3) We will investigate the other differences in skeletal muscle of goats at different ages in the future.** We think the reviewer give us several nice suggestions. Therefore, we will discuss the other differences in skeletal muscle of goats at different ages in the future.

Line 277-279, Adult goats are usually slaughtered, and kids of goats are rarely slaughtered for meat, so it makes no sense to talk about the meat quality of adult goats being better than kids'.

Response: We agree to the reviewer’s judgement that “it makes no sense to talk about the meat quality of adult goats being better than kids'”. Because we only discussed IMF content, one of the indicators of meat quality, in the current paper, we can not come to the conclusion that “the meat quality of adult goats being better than kids'”. We should just give the fact that IMF content in skeletal muscle of adult goats is higher than that in kids without mentioning the meat quality. Therefore, we deleted “indicating adult meat of Hainan black goats are better in meat quality than kid meat” Line 279 in initial version.

**About** **slaughtering kid goats for meat**. According to the reviewer’s question, we checked our paper again and found that we need add a little information about slaughtering kid goats for meat. Traditionally, people in Hainan province, the southmost province of China, like to eat kid’s meat very much and deems kid’s meat is better than adult’s meat. Thus, the comparison of the differences in skeletal muscle of goats at different ages is very necessary. Therefore, we added the following content that “such as high meat quality, earlier sexual maturity, roughage- and heat- resistance, stronger disease resistance and genetic stability. Traditionally, people lived in Hainan province likes to consume kids’ meat and they thought the meat quality of kids’ is better than that of adults’. Generally, the meat quality is associated with intramuscular fat (IMF) content [14], muscle microstructure (such as muscle fiber diameter) [15], shear force [16], drip loss after 24/36 h of death [17] and so on. To provide evidences in understanding whether the meat quality of kids’ is better than that of adults’ and ….” in the revised version (Line 65-71 in revised version).

Figure 1 to 5. The image is not clear, the files are too big to download, it takes too long to download them, the .tif files should be in compressed format.

Response: We have compressed the figures and resubmitted them again. The sizes for all of figures are compressed sharply.

I suggest the authors rewrite the paper and resubmit it, not to associate with intramuscular fat. Because there are a lot of differences in longissimus dorsi muscle between kids and adult goats.

Response: We thank you for your kind suggestion. We completely agree to your viewpoint that there are a lot of differences in longissimus dorsi muscle between kids and adult goats. In this paper, we have some considerations about the aims of this paper, which has been mentioned in the above response. Based on the considerations, we just focus on the IMF differences in skeletal muscle of goats at different ages. For the other differences in LD muscle between kids and adult goats, we will investigate their molecular basis in the future and will organize another paper to bring forth the results.

**Reviewer #2:**

The long non-coding RNAs (lncRNAs) play essential roles in intramuscular fat deposition of Animals. In this study, the author expounded which lncRNAs are associated with IMF deposition and how lncRNAs and genes play their roles in Hainan Black goats. And the results of this paper are very interesting and it is an excellent paper. However, there are still some minor problems that should be modified.

Response: We thank the reviewer for his/her positive evaluation.

1. Please add the measurement methods of meat quality indicators such as intermuscular fat and the models of the instruments used in the section of materials and methods.

Response: We have added the measurement methods of meat quality indicators in the revised version. The changed context includes, 1) Adding “meat quality measurement” into the subtitle of “Animals, sample collection and RNA isolation” in revised version (Line 80 in revised version). 2) Adding “Samples used for meat quality measurement were first divided into four pieces, and each of the pieces was used to measure an indicator of meat quality. IMF content was measured by using soxhlet extraction method (Soxhlet extractor, Shanghai, China). For muscle fiber diameter, we firstly made H.E paraffin wax section and then measured muscle fiber diameter, which was described detailed in Gu et al [19]. Muscle moisture was determined by using moisture determinator (SFY-30R, Shenzhen, China). 24h suspension water loss rate, 36h suspension water loss rate and shear force were measured according to the criteria of GB2707-2016.” (Line 89-96 in revised version).

2. The word “important” is often overused. Consider using a more specific synonym to improve the sharpness in this paper.

Response: We changed the“important” to the specific synonym. Such as to “crucial” in Line 19, to “essential” Line 20, to “key” in Line 50, to “crucial” in Line 254, to “crucial” in Line 324 of revised version.

3. The language should be improved by a native English speaker in order to eliminate grammatical and spelling errors and to conform to correct scientific English. I listed some of these errors below.

Line 24-26: Then, the lncRNA-seq and RNA-seq data were integrated analyzed to explore the IMF-related lncRNAs and genes and their potential functions. Please add the word and between integrated and analyzed.

Response: We added the word “and” between integrated and analyzed and marked in red (Line 25 in revised version).

Line 30: It is 2,079 not 2 079.

Response: We changed 2 967 to “2,967” (Line 30 in revised version).

Line 43: “their” should be changed to “a”.

Response: We changed “their” to “a” (Line 44 in revised version).

Line 48: “the exploration of” should be changed to “exploring”.

Response: We changed “the exploration of” should be changed to “exploring” (Line 48 in revised version).

Line 51: It is “et al.” not “et al”, please check it all through this paper.

Response: We changed “et al” to “et al.” (Line 52 in revised version). In addition, we checked all of it throughout this paper and added”.” at each position.

Line 62: “originated from” shoud be changed to “in”.

Response: We changed “originated from” to “in” (Line 63 in revised version)

Line 65: “Especially” shold be changed to “Primarily”, and “is” should be changed to “are”.

Response: The content has been replaced in revised version.

Line 81: “muscle” should be changed to “muscles”.

Response: We changed “muscle” to “muscles” (Line 85 in revised version).

Line 85: add a comma after analysis, and delete the word one.

Response: We have changed according to the suggestion (Line 88 in revised version).

Line 87: delete the “old of age”.

Response: We deleted the “old of age” (Line 98 in revised version).

Line 88: delete the “of age”.

Response: We deleted the “of age” (Line 98 in revised version).

Line 89: “allow” should be changed to “allowing”.

Response: We changed “allow” to “allowing”.

Line 117-118: the sentence “To identify goat annotated lncRNAs and mRNAs, we filtered the complete transcripts obtained above.” should be changed to “We filtered the complete transcripts obtained above to identify goat annotated lncRNAs and mRNAs.”

Response: We changed the sentence “To identify goat annotated lncRNAs and mRNAs, we filtered the complete transcripts obtained above.” to “We filtered the complete transcripts obtained above to identify goat annotated lncRNAs and mRNAs.” (Line 128-129 in revised version).

Line 121: delete the “that were”.

Response: We deleted the “that were”.

Line 131: “A transcript” should be changed to “As a result, a transcript”.

Response: We changed “A transcript” should be changed to “As a result, a transcript” (Line 142 in revised version).

Line 138-139: “We, then, used DEGseq to analyze the difference significances” should be changed to “Then, we used DEGseq to analyze the different significances”.

Response: We changed “We, then, used DEGseq to analyze the difference significances” to “Then, we used DEGseq to analyze the different significances” (Line 149-150 in revised version).

Line 147: deleted “of them”.

Response: We deleted “of them” of line 147 in initial version.

Line 156: “transcription of RNA” should be changed to “RNA transcription”.

Response: We changed “transcription of RNA” to “RNA transcription” (Line 167 in revised version).

Line 170: “for the analysis of” should be changed to “to analyze the”.

Response: We changed “for the analysis of” to “to analyze the” (Line 186 in revised version).

Line 184: delete the word “enough”.

Response: We deleted the word “enough” (Line 200 in revised version).

Line 188-189: “Comparing with mRNA, the average length was shorter,” should be changed to “The average length was shorter than mRNA,”.

Response: We changed “Comparing with mRNA, the average length was shorter,” to “The average length was shorter than mRNA,” (Line 204-205 in revised version).

Line 193: “Comparing” should be changed to “Compared”.

Response: We changed “Comparing” to “Compared” (Line 209 in revised version).

Line 207: “ranged” should be changed to “ranging”.

Response: We changed “ranged” to “ranging” (Line 223 in revised version).

Line 225-226: “Through GO analysis, we got 728 significantly enriched GO term” should be changed to “We got 728 significantly enriched GO terms through GO analysis, including…”.

Response: We changed “Through GO analysis, we got 728 significantly enriched GO terms” to “We got 728 significantly enriched GO terms through GO analysis, including…” (Line 242-243 in revised version).

Line 275：“allow for the retention of higher levels of water” should be changed to “allow for higher levels of water retention”.

Response: We changed “allow for the retention of higher levels of water” to “allow for higher levels of water retention” (Line 292 in revised version).

Line 279: “are” should be changed to “is”.

Response: We have deleted the sentence in revised version.

Line 290: “The exploration of” should be changed to “Exploring”.

Response: We changed “The exploration of” to “Exploring” (Line 310 in revised version).

Line 299: “which corresponded” should be changed to “corresponding”

Response: We changed “which corresponded” to “corresponding” (Line 319 in revised version).

Line 305: “identification of” should be changed to “identifying”.

Response: We changed “identification of” to “identifying” (Line 325 in revised version).

4. Please standardize the format of all references.

Response: We standardized format of all references according to the reference requirement of PLOS ONE. We changed the styles of reference 4 and 7, which we marked in red. In addition, we added a reference, [16], and then changed the serial number of the subsequently references in the revised version and marked in red.
