# Supplementary material for: Integrated analysis of lncRNA and gene expression in longissimus dorsi muscle at two developmental stages of Hainan black goats
Source: PLoS One. 2022 Oct 31;17(10):e0276004. doi: 10.1371/journal.pone.0276004 (PMC9621442; doi:10.1371/journal.pone.0276004)
Supplement: Attachment — Submitted filename: Response to Reviewers-R2.docx [file pone.0276004.s011.docx]

PONE-D-22-12097-R2

Integrated analysis of lncRNAs and genes associated with intramuscular fat in longissimus dorsi muscle at two developmental stages of Hainan black goats

PLOS ONE

**Journal Requirements:**

Please review your reference list to ensure that it is complete and correct. If you have cited papers that have been retracted, please include the rationale for doing so in the manuscript text, or remove these references and replace them with relevant current references. Any changes to the reference list should be mentioned in the rebuttal letter that accompanies your revised manuscript. If you need to cite a retracted article, indicate the article’s retracted status in the References list and also include a citation and full reference for the retraction notice.

Response: We have reviewed reference list and corrected them according to the requirement for references of PLOS ONE. The changed points are listed as follow.

(1) We deleted the doi, PMID and PMCID for each reference.
(2) We changed “indent first row” for each reference to “suspension indent”.
(3) We deleted the space after the year and the space after volume period for each reference.
(4) We deleted “and” between the two authors of reference 1.
(5) We changed the reference 24 in R1 version by a new reference in R2 version.
(6) We changed the “,” after “Genet Mol Res” to “.” for reference 19.

**Responses to Reviewers**

6. Review Comments to the Author

Please use the space provided to explain your answers to the questions above. You may also include additional comments for the author, including concerns about dual publication, research ethics, or publication ethics. (Please upload your review as an attachment if it exceeds 20,000 characters)

**Reviewer #1:**

Title: I suggest that the title should be changed to “Integrated analysis of lncRNA and gene expression in longissimus dorsi muscle at two developmental stages of Hainan black goats”

Response: We changed the full title to “Integrated analysis of lncRNA and gene expression in longissimus dorsi muscle at two developmental stages of Hainan black goats” in R2 version.

Abstract

Line 19-24, Screening the differentially expressed lncRNAs in muscle of goats of different ages was the objective of this study, but not all these differentially expressed lncRNAs are related to intramuscular fat. Thus, the objective of this study should be modified in the Abstract. The authors just changed the title, but not changed the objective and other parts related to IMF. Just as I said before, “Although the intramuscular fat (IMF) content of goats at different ages was different, there were other differences in skeletal muscle of goats at different ages, such as tenderness, water content, protein content, amino acids and fatty acids, meat color, pH, etc.”

Response: We thanked your kind suggestion. According to your suggestion, we modified the background and objective of this study. The revised abstract is listed as follow “It is deemed that meat quality of kids’ is better than that of adults’ for Hainan black goat. Generally, meat quality is affected by many indicators, such as intramuscular fat (IMF) content, muscle fiber diameter and shear force. It is indicated that long non-coding RNAs (lncRNAs) play essential roles in meat quality of goats. However, it is unclear whether and how lncRNAs and genes play their roles in meat quality of Hainan Black goats. Here, we firstly compared the meat quality between two-month-old kids (kids) and adult goats (adults). Then, the lncRNA-seq and RNA-seq data were integrated and analyzed to explore the potential functions of lncRNAs and genes. The results showed that adults' IMF content and muscle fiber diameter were extremely significantly higher than that of kids (P<0.01). For the sequenced data, average 84,970,398, and 83,691,250 clean reads were obtained respectively for Kids and adults, among which ～96% were mapped to the reference genome of goats. Through analyzing, 18,242 goat annotated genes, 1,429 goat annotated lncRNAs and 2,967 novel lncRNAs were obtained. Analysis of differential expression genes (DEGs) and lncRNAs (DELs) showed that 328 DEGs and 98 DELs existed between kids and adults. Furthermore, functional enrichment analysis revealed that a number of DEGs and DELs were mainly associated with IMF. Primarily, DGAT2 expressed higher in adults than that in kids and CPT1A expressed higher in kids than that in adults. Both of them were overlapped by DEGs and targets of DELs, suggesting the two DEGs and the DELs targeted by the two DEGs might be the potential regulators of goat IMF deposition. Taken together, our results provide basic support for further understanding the function and mechanism of lncRNAs and genes in meat quality of Hainan black goats.” Line 19-38 in R2 version.

How do you know that the differentially expressed lncRNAs that you screened are only related to IMF and not related to other indicators of meat quality? The differentially expressed lncRNAs that you screened would be associated to any different indicator of goat skeletal muscle at different ages. Therefore, it is not logical to emphasize only IMF in this study.

Response: Thank you for your constructive advice. In fact, only some of the differentially expressed lncRNAs that we screened are related to IMF. There are still some differentially expressed lncRNAs that are related to muscle fiber diameter and shear force. Therefore, we changed our statements without only emphasizing IMF. Some of the changes are listed as follow.

(1) We changed the objective of this study of R1 version in Line 19-24 to “It is deemed that meat quality of kids’ is better than that of adults’ for Hainan black goat. Generally, meat quality is affected by many indicators, such as intramuscular fat (IMF) content, muscle fiber diameter and shear force. It is indicated that long non-coding RNAs (lncRNAs) play essential roles in meat quality of goats. However, it is unclear whether and how lncRNAs and genes play their roles in meat quality of Hainan Black goats. Here, we firstly compared the meat quality between two-month-old kids (kids) and adult goats (adults). Then, the lncRNA-seq and RNA-seq data were integrated and analyzed to explore the potential functions of lncRNAs and genes. The results showed that adults' IMF content and muscle fiber diameter were extremely significantly higher than that of kids (P<0.01)” Line 19-27 in R2 version.

(2) We changed “IMF deposition” Line 38 in R1 version to “meat quality” Line 38 in R2 version.

(3) We changed “Considering the significant differences in IMF content” Line 228 in R1 version to “Considering the significant differences in IMF content, muscle fiber diameter and shear force” Line 222-223 in R2 version.

(4) We changed “IMF deposition” Line 230 in R1 version to “meat quality” Line 225 in R2 version.

(5) We added a sentence of “The results showed that DELs obtained in this study are mainly related to IMF.” Line 249 in R2 version.

(6) We modified the two paragraphs of Line 289-296 and Line 369-377 in R1 version according to your suggestions below. The revised contents are listed in Line 287-294 and Line 368-377 in R2 version.

(7) We modified multiple statements of the paragraph in Line 321-337 in R2 version.

Line 188-189, Why are these lncRNAs not associated with shear force or muscle fiber diameter? In addition, the authors did not determine the meat color, pH, the profile of amino acids and fatty acids of goat muscle at different ages. Did these indicators of meat quality differ as well?

Response: We think the reviewer proposed a nice question. We indeed associated the DEGs and DELs with shear force and muscle fiber diameter in R2 version. For example, we added a sentence of “In addition, we obtained some DEGs, including *PAX3, IGF1, TGFBR3, HOXA4, IGF2, TGFBI, MYF6, and JUNB*, were associated tightly with muscle fiber diameter and shear force” Line 249-251 in R2 version. In addition, we added a sentence of “We also found that some DEGs were associated with muscle fiber diameter and shear force, such as *PAX3, IGF1, TGFBR3, HOXA4, IGF2, TGFBI, MYF6, and JUNB*” at discussion section of Line 329-331 in R2 version.

Finally, we are investigating the differences for meat color, pH, the profile of amino acids and fatty acids of goat muscle at different ages according to your previous suggestion in R1. However, we have not got the results yet.

Line 289-296, This paragraph needs to be appropriately revised, do not only focus on the IMF. Other meat quality indicators are also different between two-month-old kids and adult goats.

Response: We have modified the paragraph according to your suggestion. The revised paragraph is listed as follow “With the improvement of people's living standard, more nutritious and delicious meat is required. Many factors could affect meat quality, including age, IMF, nutrition, genotype, type of muscle, feeding regime, and handling and slaughtering conditions [23]. It is reported that lower redness and shear force were observed in the breast and leg meat of 70-day-old geese when compared with 90- or 120-day-old geese [24]. In addition, the greater IMF of meat in Iberian wild red deer may render it more palatable [25]. In this study, IMF and muscle fiber diameter in adult LD muscle were extremely significantly higher than that in kid, while shear force in adult LD muscle was significantly higher than that in kids” Line 287-294 in R2 version.

Line 369-377, This paragraph also needs to be appropriately revised, do not only focus on the IMF.

Response: We have modified the paragraph according to your suggestion. The revised paragraph is listed as follow “In summary, much higher IMF content, muscle fiber diameter and shear force in adults than that in kids were found in LD muscles of Hainan black goats. We obtained a number of DEGs and DELs between the LD muscles of adults and kids, which may provide valuable resources for investigating the molecular mechanism of the differences in LD muscles between kids and adults of Hainan black goats. Our findings also showed that DEGs and DELs were tightly associated with meat quality of Hainan black goats. Especially, *DGAT2 and CPT1A* genes, two key genes in fat metabolism, may play crucial roles in IMF content and meat quality of Hainan black goats because they were covered by DEGs and targets of DELs. Overall, our findings may serve as fundamental resources for a deeper understanding in the regulation of goat meat quality” Line 368-377 in R2 version.

**Response to upload your figure files to the Preflight Analysis and Conversion Engine (PACE):**

While revising your submission, please upload your figure files to the Preflight Analysis and Conversion Engine (PACE) digital diagnostic tool, https://pacev2.apexcovantage.com/. PACE helps ensure that figures meet PLOS requirements. To use PACE, you must first register as a user. Registration is free. Then, login and navigate to the UPLOAD tab, where you will find detailed instructions on how to use the tool.

Response: We have uploaded our figure files to PACE digital diagnostic tool and downloaded the corrected figure files. The figures that uploaded to submission system of PLOS ONE for R2 version are the figures corrected by PACE.
